# Annexin 1 and Melanocortin Peptide Therapy for Protection Against Ischaemic-Reperfusion Damage in the Heart

**DOI:** 10.1100/tsw.2006.196

**Published:** 2006-08-26

**Authors:** F.N.E. Gavins, G. Leoni, S.J. Getting

**Affiliations:** ^1^ Centre for Biochemical Pharmacology, The William Harvey Research Institute, London, EC1M 6BQ, UK; ^2^ School of Biosciences, Department of Human and Health Sciences, University of Westminster, London, W1W 6UW, UK

**Keywords:** annexin 1, melanocortin, neutrophil, migration, anti-inflammatory, macrophage, heart, ischaemic-reperfusion, cytokine, chemokine

## Abstract

Cardiovascular disease is a major cause of mortality within the western world affecting 2.7 million British people. This review highlights the beneficial effects of naturally occurring hormones and their peptides, in myocardial ischaemic-injury (MI) models, a disease pathology in which cytokines and neutrophils play a causal role. Here we discuss two distinct classes of endogenous peptides: the steroid inducible annexin 1 and the melanocortin peptides. Annexin 1 and the melanocortins counteract the most important part of the host inflammatory response, namely, the process of leukocyte extravasation, as well as release of proinflammatory mediators. Their biological effects are mediated via the seven transmembrane G-protein-coupled receptors, the fMLP receptor family (or FPR), and the melanocortin receptors, respectively. Pharmacological analysis has demonstrated that the first 24 amino acids of the N-terminus (termed Ac2-26) are the most active region. Both exogenous annexin 1 and its peptides demonstrate cardioprotectiveness and continuing work is required to understand this annexin 1/FPR relationship fully. The melanocortin peptides are derived from a precursor molecule called the POMC protein. These peptides display potent anti-inflammatory effects in human and animal models of disease. In MI, the MC3R has been demonstrated to play an important role in mediating the protective effects of these peptides. The potential anti-inflammatory role for endogenous peptides in cardiac disease is in its infancy. The inhibition of cell migration and release of cytokines and other soluble mediators appears to play an important role in affording protection in ischaemic injury and thus may lead to potential therapeutic targets.

## MYOCARDIAL ISCHAEMIA REPERFUSION

Cardiovascular disease is the leading cause of death in people over 65 years of age. At least one-third of all cardiovascular disease is attributable to five risk factors: smoking, alcohol, high blood pressure, high cholesterol, and obesity. Indeed, it is believed that over a billion people will die from cardiovascular disease in the first half of the 21$^{st}$ century (Anthony Rogers, Clinical Trials Research Unit, University of Auckland, NZ, 2004). The financial costs of treating such people are rapidly increasing and, therefore, a greater understanding of the disease should lead to more effective treatments, a reduction in finance, and an increase in "peace of mind".

Ischaemia reperfusion (IR) is implicated in many different cardiac conditions, including thrombolysis, angioplasty, and coronary bypass surgery, which are frequently used to establish the blood reflow and minimise the damage to the heart due to severe myocardial infarction. In a clinical situation of myocardial IR (MIR), surgeons are able to intervene at certain points, such as at the time of coronary catheterisation or entry into emergency care facility[1].

Early induction of reperfusion is an absolute prerequisite for the survival of ischaemic myocardium[2] and can be an effective way to prevent the progression of ischaemic cell necrosis. However, reperfusion is a double-edged sword, and the restoration of blood flow into a previously ischaemic zone is not always totally beneficial. Although a significant amount of injury occurs due to ischaemia itself, a great deal of damage also occurs to the myocardium because of reperfusion,

During myocardial reperfusion, several mechanisms mediate vascular injury. The production of oxygen free radicals (OFRs) is increased by a number of different factors, such as (1) mitochondrial respiration (ischaemia changes the redox state and promotes xanthine), (2) an increase in activated neutrophils, and (3) up-regualtion of xanthine oxidase activity. This activates leukocytes, induces lipid peroxidation, and increases vascular permeability[3]. Several models of MIR have shown there to be a sudden efflux of oxygen metabolites (e.g., superoxide radical, hydroxyl radical, and peroxynitrite) when oxygen is allowed back into a previous ischaemic area[4]. These oxygen metabolites are extremely reactive and cause irreversible damage to cell membranes; the addition of superoxide dismutase reduces OFR concentrations in reperfused myocardium[5]. In ischaemic-reperfused hearts, depression of contractile function, arrthymias, change in gene expression, and loss of adrenergic pathways have also been observed, partly due to increased OFRs. It still remains controversial whether OFRs increase during ischaemia, although it is accepted that their formation occurs during reperfusion. There is also an activation of complement[6], decreases in nitric oxide production[7], and increases in leukocyte-endothelial cell interactions, which can cause capillary plugging and the "no-reflow" phenomena[3]. Some studies have shown that active capillary plugging, i.e., the ability of pericytes (distributed along the capillaries) to induce constriction in these capillaries by contracting cell processes that partially envelope the capillary[8], may cause the reduction in cardiac capillary cross-sectional dimensions in IR injury. This idea still remains controversial, but it does appear that the capillary bed may play a much greater role in the local control of blood flow than was once previously thought.

Although there is a reduction in infarct size when neutrophils are depleted using antineutrophil antiserum[9] or leukopak filters[10], the specific role of neutrophils still remains unclear. An abundance of experimental evidence points to an important role of the selectins and adhesion molecules in neutrophil recruitment during inflammation. P-selectin antibodies, e.g., PB1.3, have been shown to reduce infarct size and associated risk, along with attenuating endothelial dysfunction[11,12]. Also, a recombinant analogue of the major PSGL-1 has been shown to be protective in a feline model of coronary occlusion and reperfusion[13]. L-selectin monoclonal antibody also reduces myocardial necrosis by approximately 50% in a feline model[14]. E-selectin KO mice also have smaller infarct sizes post-IR[15]. The blockade of ICAM-1[16] or CD18[17] partially attenuates myocardial reperfusion injury. However, it is clear that myocardial injury *in-vivo* is not dependent on any one adhesion molecule.

Indeed inflammation is now thought to play a serious role in both the initiation and progression of cardiovascular disease. Many different animal studies and clinical trials have investigated the risk factors associated with cardiovascular disease, such as obesity, hypertension, and smoking, and have provided clear evidence to suggest that various aspects of the inflammatory response associated with these risk factors do heighten the damaging effect. Much evidence has been provided by the investigation of the endothelium, as endothelial dysfunction is an early event and expression of the adhesion glycoproteins by activated endothelial cells is a rate-limiting step in the recruitment of inflammatory cells[18].

However, despite the growing body of evidence that inflammation plays a key role in the progression of cardiovascular disease, many clinical trials have failed, e.g., to date, no antineutrophil clinical trials have been successful. Trials using humanised anti-CD18 mAbs (FESTIVAL[19]) and LIMIT-AMI[20] have been administered to patients with myocardial infarction, but no effect was observed in infarct size.

## CURRENT THERAPIES

Table 1 demonstrates the vast choice of medication that is available to treat and manage cardiovascular disease.

**TABLE 1**
**Medication for the Treatment and Management of Cardiovascular Disease (Adapted from World Health Organisation)**

| Medication Type | Used For | Examples |
|---|---|---|
| Antiplatelet agent | Prevention of blood clots | Statins |
| Anticoagulant or blood thinner | Prevention of blood clots. Used in patients with atrial fibrillation and after heart valve replacement surgery. | Warfarin |
| Vasodilator | Blood vessel relaxation. Prevention and relief of angina. | Nitrates |
| Diuretic | Removes excess water from the body and prevents build up. Lowers blood pressure. Used for high blood pressure and heart failure. | Furosemide<br>Thiazides |
| Calcium-channel blockers | Relaxes blood vessels and lowers blood pressure. Used for high blood pressure and angina. | Nifedipine |
| Beta-blocker | Slows the heart rate and increases force of heartbeat. Used for high blood pressure and angina. | Atenolol |
| Angiotensin-converting enzyme (ACE) inhibitor | Relaxes blood vessels and reduces strain on the heart. Used for high blood pressure. | Enalapril |
| Centrally acting antihypertensive | Lowers blood pressure by acting on the brain. | Methyldopa |
| Angiotensin II receptor blocker (ARB) | Dilates blood vessels and lowers blood pressure. | Cadesartan |
| Cardiac glycoside | Increases the strength of heart muscles and helps heart pump blood. Used for heart failure. | Digoxin |
| Blood cholesterol–lowering agent | Lowers cholesterol level. | Statins |
| Biguanide | Helps body cells to take in sugar. Used for diabetes. | Metformin |
| Sulfonylurease | Increases insulin production. Used for diabetes. | Glibenclamide |

However, there are several lines of approach that are currently being investigated for the treatment and management of cardiovascular disease. Some of these therapies are as follows:

1. Creation of new drugs that increase the levels of high-density lipoproteins (HDLs), which in-turn will lower heart disease.
2. Development of angiogenesis drugs, which will aid the growth of new arteries on the heart, and thus reduce the need for bypass surgery.
3. Develop antiglycosylation therapies for the prevention of cross-linking that weakens aging heart muscle, and reduce diabetic risk factor.
4. Investigation of combining nicotinic acid (a potent agent for increasing HDL cholesterol and reducing LDL cholesterol) with a statin, which may help to reduce coronary heart disease[21].
5. Gene therapy as a potential strategy for treating cardiovascular disease; combining this therapy with cell therapy and tissue engineering, gene silencing, and the targeting of genes in the vascular wall and the myocardium.

Another line of interest is the study of inflammatory markers and mediators that may lead to potential therapeutic strategies and drug targets, with the development of compounds that may have fewer side effects. The potential anti-inflammatory role for endogenous peptides in cardiac disease is in its infancy. The inhibition of cell migration and release of cytokines and other soluble mediators appears to play an important role in affording protection in ischaemic injury and thus may lead to potential therapeutic targets. This review will focus on the beneficial effects of two different types of endogenous peptides: annexin 1 and melanocortin.

## ANNEXIN 1

Glucocorticoids are synthesised by the adrenal cortex and endogenously released to reduce the inflammatory response, e.g., they can act as potent anti-inflammatory drugs by inhibiting phospholipase $A_2$ ($PLA_2$) activity, as well as cyclooxygenase-2 (COX-2) and inducible nitric oxide species (iNOS) expression, thus preventing autocoid release and function[22]. Glucocorticoids are also potent inhibitors of neutrophil tissue damage[23].

In the late 1970s, a new protein was discovered and characterised by its ability to quash inflammatory mediators of the eicosanoid family by suppressing the activity of the enzyme $PLA_2$. The action on arachidonate and eicosanoid release *in vitro*[24] was accompanied by an inhibitory effect in experimental models of inflammation *in vivo* (e.g., $TXA_2$ release from perfused guinea pig lungs[25]. This novel protein is now termed “annexin 1”.

Annexin 1 is a 37-kDa (consisting of 346 amino acids; Fig. 1) member of a superfamily of proteins (there are 13 mammalian annexins). The larger portion of this protein is formed by a 70-amino-acid motif, which is repeated four times, which are then grouped together to form a globular structure with a convex and concave face. Binding of $Ca^{2+}$ and phospholipids to annexin is mediated through a type II and type III $Ca^{2+}$-binding site within the core domain, and $Ca^{2+}$ bound within these sites serves as a platform through which they interact with peripheral membrane phospholipids. A unique N-terminus attached to this core domain varies in length between the different members of the superfamily (Fig. 1).

In murine peripheral leukocytes, annexin 1 concentrations are highest in neutrophils and lowest in lymphocytes, with an intermediate amount in monocytes[26]. The glucocorticoid dexamethasone (DEX) increased annexin 1 expression in neutrophils and monocytes. In human leukocytes, annexin 1 is found in largest amounts in neutrophils and monocytes, with low and varying amounts in T-lymphocytes[27], and no expression in B lymphocytes. The largest pool in lymphocytes is in CD56+ natural killer cells[28].

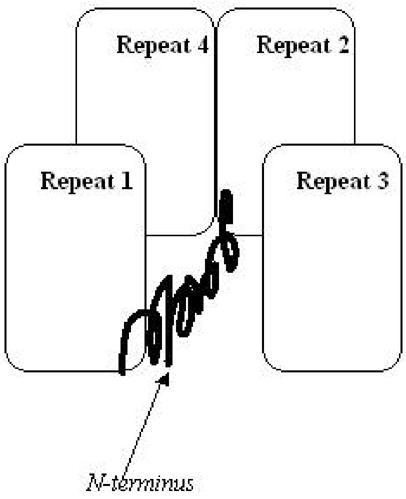

**FIGURE 1.** A schematic representation of annexin 1 and its N-terminal domain. The four repeats of annexin 1 are shown in which repeat 1 and 4 and repeat 2 and 3 are paired. The N-terminal domain is attached to repeat 1.

Since annexin 1 is most abundant in neutrophils, it is considered to be the most likely candidate to mediate calcium-dependent membrane fusion during phagocytosis and/or exocytosis of neutrophils. When neutrophils adhere to endothelial cells, annexin 1 is externalised from its storage site (gelatinase granules) to the cell surface[29] where the endogenous protein acts in an auto/paracrine manner to inhibit the process of leukocyte diapedesis. This reduction in inflammation by annexin 1 is achieved in different ways, such as reduced paw oedema, decreased PMN migration, antipyretic effects, and an antiendotoxic action. *In vitro*, this effect is observed when neutrophils adhere to monolayers of endothelial cells, annexin 1 is externalised onto the cell surface, down-regulating the cell's emigration and thus activating other secondary pathways.

Following migration into the tissue, annexin 1 is cleaved. Perretti al.[30] used anti-annexin 1 sheep serum, raised against the full length of annexin 1 to investigate this aspect. Intravascular neutrophils adherent to the endothelium retained cell surface annexin 1 in its intact form (i.e., 37 kDa), whereas annexin 1 in migrated cells was mostly cleaved (i.e., 34 kDa) and localised in large endosome-like vacuoles. This study suggests that the immunoreactivity of the vacuoles of extravasated neutrophils is due to internalised intact annexin 1. Western blotting analysis also shows annexin 1 cleavage during inflammation. A "lipocortinase" has been proposed to be responsible for this cleavage, being selectively activated during the process of neutrophil adhesion/emigration[31].

## Annexin 1 and Its Peptides

Peptides (Fig. 2) derived from the N-terminus of annexin 1, e.g., Ac2-26, Ac2-12, and Ac2-6, have been used in many studies[32]. The peptide spanning the first 24 amino acids of annexin 1, termed Ac2-26, has been shown to mimic the human recombinant annexin 1 for its ability to inhibit neutrophil migration in inflammatory models. Peptide Ac2-26 is approximately 200 times less potent than the parent compound, although the dose-response curve is parallel, implying similar efficacy[33]. The technique of intravital microscopy has been used to demonstrate that both annexin 1 and peptide Ac2-26 are able to inhibit blood-borne leukocyte extravasation. To be more specific, annexin 1 and its mimetic peptide demonstrated that only the fate of adherent leukocytes was affected, whereas rolling and the extent of leukocyte adhesion were unaffected[34,35].

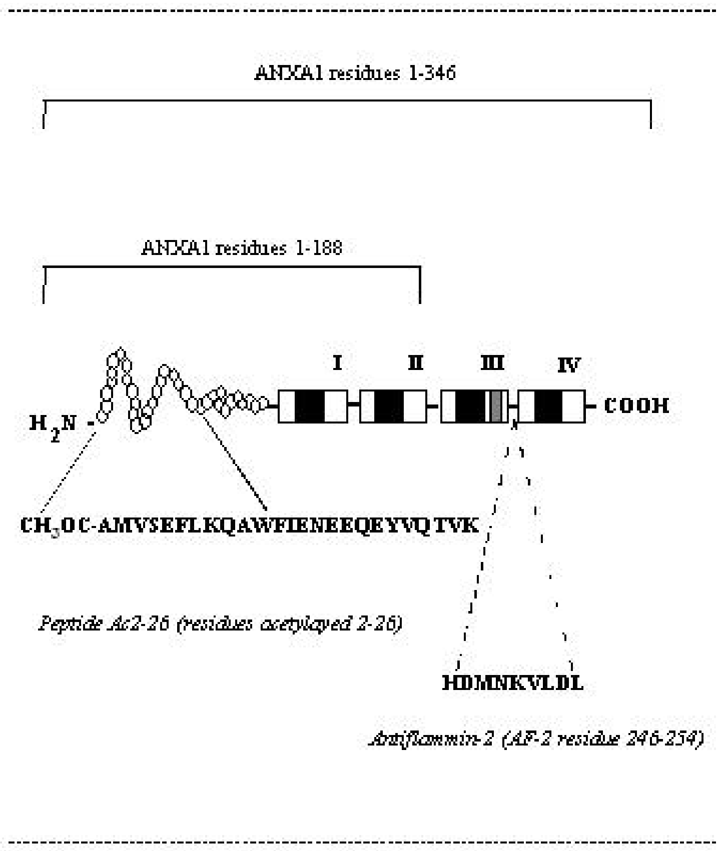

**FIGURE 2.** A schematic representation of annexin 1 (ANXA1) and peptides derived from the sequence.

Other peptide sequences have been derived from the core of annexin 1; these are termed the antiflammins[36] (Fig. 2). One encompasses amino acids 39-47 (MQMKKVLDS) of an uteroglobin sequence and is termed antiflammin 1 (AF-1), and a second from the 246-254 (HDMNKVLDL) of annexin 1, termed antiflammin 2 (AF-2). The antiflammins also inhibit cytosolic phospholipase $A_2$ ($cPLA_2$) in a concentration-dependent manner *in vitro,* and reduce oedema formation following local injection of carrageenin into the rat paw[36]. However, some authors have found it difficult to repeat the inhibitory effect on $cPLA_2$ activity[37,38], raising doubts about the real effectiveness of the antiflammins as enzyme inhibitors. Nonetheless, antiflammins have been shown to inhibit leukocyte adhesion to human leukocytes and coronary artery endothelial cells, by attenuating activation-induced upregulation of CD11/CD18 on leukocytes[39].

## Annexin 1 and Its Receptors

In 2000, Walther and colleagues confirmed, using *in vitro* techniques, the antimigratory effects of peptide Ac2-26, and linked Ac2-26 to the receptor for formylated peptide (FPR)[40]. This group showed that Ac2-26 provoked transient changes in intracellular calcium and L-selectin shedding from the plasma membrane, which were prevented by the addition of FPR antagonists, i.e., the butoxyl-carbonyl (or Boc) derivatives.

The FPR is a G-protein-coupled receptor found mainly on leukocytes. It is one of the best-studied chemoattractant receptors and, like all other chemoattractant receptors, FPR produces a range of responses on stimulation, e.g., stimulation of migration and, at higher doses, opening calcium channels, triggering with exocytosis and a respiratory burst[41].

Two human genes that encode specific FPR subtypes have been cloned; they are termed FPR1 and FPRL1 (FPR-like 1, also referred to as FPRH2, and a functional lipoxin $A_4$ [$LXA_4$] receptor or ALXR). Both are present on human neutrophils and monocytes, and FPRL1 is also present on epithelial cells[42]. FPR binds fMLP with approximately 1000-fold higher affinity than FPRL1, resulting in calcium mobilisation and subsequent neutrophil activation. FPRL1 shares approximately 69% amino acid identity with FPR, particularly in the signalling domain and thus it has been suggested that FPR and FPRL1 are likely to transduce the same signal downstream of the receptor, but to be activated by different ligands. Various agonists that preferentially bind and activate FPRL1 have been identified, e.g., the HIV-derived peptide, V3[43]. FPRL1 also transduces anti-inflammatory signals, as is the case with $LXA_4$. Construction and screening of random peptide libraries is a useful approach to developing biologically active agents. Klein et al. isolated a number of small peptide sequences that reacted with FPR and FPRL1[44], e.g., MMK-1 (LESIFRSLLFRVM), which is one of the most potent FPRL1-specific agonists identified so far. FPRL2 has been identified by cross-hybridisation, but as of yet its function remains unclear[45].

## *Murine FPR Family*

Having described the human FPR family, the plot thickens considerably in rodents[46]. Currently, there appear to be eight structurally related genes: *Fpr, Fpr-rs1, Fpr-rs2, Fpr-rs3, Fpr-rs4, Fpr-rs5*[47], *Fpr-rs6*, *Fpr-rs7*[48], and possible three distinct proteins[47]. *Fpr* is the orthologue of human FPR and Gao et al. suggested that the human FPRL1 gene consists of both *Fpr-rs1* (which appears to be the murine receptor for $LXA_4$[49]) and *Fpr-rs2* (which is similar to human FPRL1 and FPRL2[50] in the mouse). Wang and colleagues also reported the presence of another murine receptor for $LXA_4$[48]. Mouse *FPR* displays a significantly lower affinity for its agonist, fMLP, such that micromolar concentrations of this formulated tripeptide are required to activate it[50].

The above suggests that the water is muddy for mouse FPRs. It is not clear whether this complex system is purely due to a problem in nomenclature by the various groups working in the field. Also, as of yet, not all these genes appear to be expressed as proteins and it remains to be seen whether this will happen. Species similarities are now being noted, e.g., a rat $LXA_4$ receptor has been cloned[51], which shares 74 and 84% amino acid homology with human and mouse orthologues.

## Annexin 1 and MIR

Annexin 1 has been shown to be cardioprotective in both rat and mouse MIR models[32,34,52]. Indeed this cardioprotective action displayed by annexin 1 and its N-terminus–derived peptides appears to be specific, as the structurally related annexin 5 did not reduce ischaemic damage in a rat model[32]. The expression of endogenous annexin 1 in this MIR model demonstrated that hearts from sham or naïve animals did not express annexin 1, as expected[53]. However, as seen in other inflammatory conditions[54,55], tissue infiltration brings about annexin 1 expression. In IR treated hearts, annexin 1 appeared as a characteristic doublet, with bands being found at 34 and 37 kDa. When rats were treated with Ac2-26, the doublet appeared reduced: the 37-kDa band being stronger than 34 kDa.

The enzyme responsible for this catabolism of annexin 1 is unclear, but the 34-kDa fragment lacks anti-inflammatory activity[56]. It may be that peptide Ac2-26 may compete with the intact form of annexin 1 at the enzyme level, thus reducing endogenous annexin 1 catabolism, which may help to explain why Ac2-26 displays cardioprotective action. It is also true, though, that peptide Ac2-26 may reduce the total amount of annexin 1 by reducing leukocyte extravasation, thereby indirectly reducing the extent of protein degradation detected in the inflamed tissue.

A similar effect was observed in the murine MIR model[34], in which peptide Ac2-26 was able to inhibit the effect of IR damage. By using FPR null mice, is was possible for the first time to investigate this effect further and demonstrate that the cardioprotective effect was due to an involvement of a Boc2 sensitive receptor other than the FPR.

Other members of the annexin superfamily have been investigated in relation to cardiovascular implications. During contractile dysfunction in congestive heart failure, altered levels of intracellular calcium ions have been found[57]. The annexins are a family of $Ca^{2+}$-binding proteins that are abundant in the heart (but not annexin 1) and thus may play a role in cardiac excitation-contraction coupling (which is often used to elucidate the intracellular mechanisms associated with contractile dysfunction in congestive heart failure). Matteo et al. demonstrated that alterations in the intracellular localisation of annexins (focussing on annexins 4, 5, and 6), along with up-regulation of annexins 5 and 6 in failing heart cells, suggested differential regulation of these annexins[58]. No data were provided about annexin 1, so it is not clear as to whether this particular $Ca^{2+}$ regulatory protein is involved in cardiac excitation-contraction coupling. However, some evidence suggests that the intracellular mechanism of the actions of annexin 1 may be linked to alterations in $Ca^{2+}$ handling, probably at the level of release from cytosolic stores rather than influx into the cell[59,60], similar to the actions of the glucocorticoid DEX[61].

## MELANOCORTINS

Melanocortins are ancient peptides little changed throughout evolution[62] and were first identified in common ancestors of lampreys and gnathostomes 700 million years ago[63]. These peptides are derived from a larger precursor molecule known as the pro-opiomelanocortin (POMC) protein and have been detected in the hypothalamus, pituitary, and periphery (including the immune system, spleen, lung, melanocytes, and the gastrointestinal tract[64]). The melanocortins all share a common amino acid sequence, His-Phe-Arg-Trp (HFRW), a sequence required for receptor binding, activation, and mediating their biological effects. The POMC protein contains three main domains: the N-terminus region, which contains γ-MSH, the central highly conserved $ACTH_{1-39}$ sequence, with α-MSH at its N-terminus, and the C-terminal β-lipotropin, which can be cleaved to generate β-endorphin[65]. The POMC protein is cleaved into its biologically active products following proteolytic cleavage by prohormone converting (PC) enzyme and carboxypeptidases between two pairs of basic amino acid residues (-Lys-Lys, -Arg-Lys-, -Arg-Arg-, -Lys-Arg-). There are seven members of the PC family, including PC1/3, PC2, furin/PACE, PACE4, PC4, PC5/6, and PC7/SPC7/LPC/PC8[66], with PC1 cleaving POMC protein into $ACTH_{1-39}$ and β-lipotropin together with low concentrations of β-endorphin, whilst PC2 cleaves POMC protein into β-endorphin and β-MSH[67].

### Melanocortin Receptors

Melanocortins exert their biological effects by binding to G-protein-coupled seven transmembrane receptors (GPCRs). They are the smallest family of GPCRs due to the fact that they have a small second extracellular loop and short amino and carboxy terminal ends[68]. Five melanocortin receptors (MCR) have been cloned and termed MC1R to MC5R; they are positively coupled to adenylylate cyclase with agonism leading to cAMP accumulation within the target cell. They have been shown to have a high sequence homology and can be detected in many different tissues[68].

- **MC1R** — This receptor displays many different functions, including pigmentation and antipyretic and anti-inflammatory actions. Expression occurs in a number of tissues including melanocytes, RAW264.7[69] and THP-1 macrophage (MØ) cell lines[70], human monocytes[71], neutrophils[72], endothelial cells[73], fibroblasts[74], mast cells[75], and lymphocytes[76]. Although predominately a peripheral receptor, it has been detected in rat and human brains in neurons of the periaqueductal gray substance[77].

- **MC2R** — The MC2R is unique since only $ACTH_{1-39}$ will activate this receptor with no biological efficacy with the melanocortin peptides. Activation of the receptor leads to a regulation in the release of steroids by the adrenal cortex essential for promotion of steroidgenic enzymes[78]. Although it is only thought to be expressed on the adrenal gland, expression has also been detected on adipocytes of mice[79], but not human[80]. Given this expression, there is the possibility that a role in metabolism might exist.
- **MC3R** — Expression occurs in the central nervous system, peripheral tissues, and immune cells, with initial studies highlighting expression in brain, gut, and placenta, but no detection in the adrenal gland or melanocytes[81]. MC3R has been postulated to be involved in modulating energy metabolism, since in MC3R null mice there is an increased fat mass and higher ratio of weight gain to food intake[82]. A role in modulating the host inflammatory response has been proposed with identification of message and protein for the receptor on peritoneal[83,84,85] and knee joint MØ[86]. Activation of resident MØ has been shown to lead to an initial inhibition of proinflammatory cytokines and chemokines, whilst at later time-points, the induction of anti-inflammatory cytokines and proteins, such as heme-oxygenase 1[87]. Finally, a role in mediating the protective effects of the melanocortins in IR injury exists[88,89]. Thus, MC3R could be proposed as a fine tuner of specific mechanisms operating during inflammation, cardiovascular function, and energy metabolism.
- **MC4R** — This receptor is unique given that it is solely expressed within many regions of the brain, including the hypothalamus, spinal cord, and cortex[90]. Most interest from the pharmaceutical industry has focused on its role in controlling food intake and energy expenditure, and could be an exciting target for controlling obesity. Other potential applications are in modulating erectile dysfunction[91] and pain[92].
- **MC5R** — Like the MC2R, MC5R is solely found in the periphery being detected in liver, lung, thymus, testis, ovary, mammary glands, fat cells, bone marrow, skin, skeletal muscle, stomach, and duodenum[68]. It is also expressed in B[93] and T[94] lymphocytes, suggesting a role in immune regulation.

## Melanocortins and MIR

Targeting of inflammatory processes, such as inhibiting proinflammatory cytokines and neutrophil migration, could be beneficial in treating this disease pathology, given the causal role that leukocytes and cytokines play in myocardial ischaemia (MI). Melanocortins are potent anti-inflammatory agents inhibiting leukocyte migration and release, and actions of cytokines and chemokines[95], therefore, they have been investigated in this pathology. Both murine and rat models of MI have been used to demonstrate the protective effects of the melanocortins. Utilising a rat model of short-term ischaemia (5 min), caused by ligature of the left anterior descending coronary artery, the parent hormone $ACTH_{1-24}$ and the potent nonselective agonist NDP-$\alpha$-MSH were effective in significantly reducing incidences of arrhythmias, lethality, and free radicals in the blood[96].

Following these initial observations, pharmacological, genetic, and molecular approaches have been undertaken to identify the receptor(s) involved in mediating this protection. To rule out a role for the MC2R expressed on the adrenal gland, a surgical approach was undertaken with rats being adrenalectomised. Following this, the protective effects of the melanocortins were still evident, indicating a potential role for other melanocortin receptors.

To try to dissect the role played by individual receptors, a panel of pharmacological tools have been used, including the selective MC1R agonist MS05[97] and selective antagonists directed at the MC4R (HS014)[98] and MC5R (HS059)[88]. MS05 failed to exert a protective effect in this model, whilst the antagonists did not attenuate the protective actions of $ACTH_{1-24}$. These data would indicate that a role for the MC1R, MC4R, and the MC5R could be excluded. Given these findings, the potential role for MC3R was evaluated using the MC3/4R antagonist SHU9119, which attenuated the protective effect, suggesting an involvement of MC3R in affording protection within the heart[88]. To try to dissect the role played by the MC3R and to confirm its pivotal role in affording protection, further pharmacological manipulation with compounds that show a selectivity towards MC3R have been utilised. Protective effects were observed with $\gamma_2$-MSH[84,86] and [D-TRP$^8$]$\gamma_2$-MSH[99], which reduced the incidence of ventricular tachycardia, fibrillation, and death, as well as an increase in free radical blood levels and fall in arterial pressure[100]. All these studies highlighted a role for MC3R utilising pharmacological rather than genetic or molecular approaches.

Recently, the cellular target for the MC3R within the heart and also the effect of the MC3/4R agonist MTII in a more chronic model of IR injury have been evaluated looking at 24-h postreperfusion. RT-PCR and western blotting highlighted message and protein for the MC3R in mouse and rat hearts; this expression was unaltered following IR. Electron microscopy showed immunogold labelling of MC3R on heart MØ, but not fibroblasts or cardiomyocytes with a punctuate distribution of receptors on the cell surface; the identification of the molecular target was further substantiated i*n vivo*. Administration of the MC3/4R agonist MTII attenuated mouse heart 2-h reperfusion injury by ~40%, an effect prevented by the mixed MC3/4R antagonist SHU9119, but not by the selective MC4R antagonist HS024. To rule out a role for the MC1R, the recessive yellow (e/e) mouse, bearing a mutated (inactive) MC1-R[101], was used with MTII, displaying a fully protective effect. Given the protective effects of melanocortin peptides in MIR, biochemical markers of inflammation have been monitored to see if they are modified by MTII. MTII reduced markers of systemic and local inflammation, including cytokine contents (interleukin-1 and KC) and myeloperoxidase activity. MTII has more importantly been shown to be effective when given at the beginning of the reperfusion period and after delayed myocardial damage as measured 24-h postreperfusion, thus suggesting a beneficial effect in acute and delayed heart reperfusion injury. These studies highlight a previously unrecognised protective role for MC3R activation and may open up new avenues for therapeutic intervention against heart and possibly other organ IR injury[89]. Table 2 summarises these findings.

**TABLE 2**
**Melanocortin Peptides Effects in Myocardial-Reperfusion Injury**

| Peptide Treatment | Observed Effects | References |
|---|---|---|
| $ACTH_{1-24}$ | Reduction in incidence of arrhythmias and lethality. Total abrogation in ischaemia-reperfusion injury induced free radicals. | [96] |
| | Reduced ventricular tachycardia, fibrillation, and lethality. Protective effects not blocked by HS014 or HS059, but abrogated by SHU9119. | [88] |
| NDP-$\alpha$-MSH | Reduction in incidence of arrhythmias and lethality. Total abrogation in ischaemia-reperfusion injury induced free radicals. | [96] |
| MS05 | Unable to prevent damage | [88] |
| $\gamma_2$-MSH<br>[D-TRP$^8$]$\gamma_2$-MSH | Reduction in incidence of ventricular tachycardia, fibrillation, and lethality | [100] |
| MTII | Reduced ischaemia at 2 and 24 h, effective when given at start of reperfusion. MC3R mRNA and protein detected in heart MØ. Inhibition of IL-1, KC, and MPO levels. All parameters inhibited in recessive yellow e/e mice. Protective effects blocked by SHU9119, but not by HS024. | [89] |

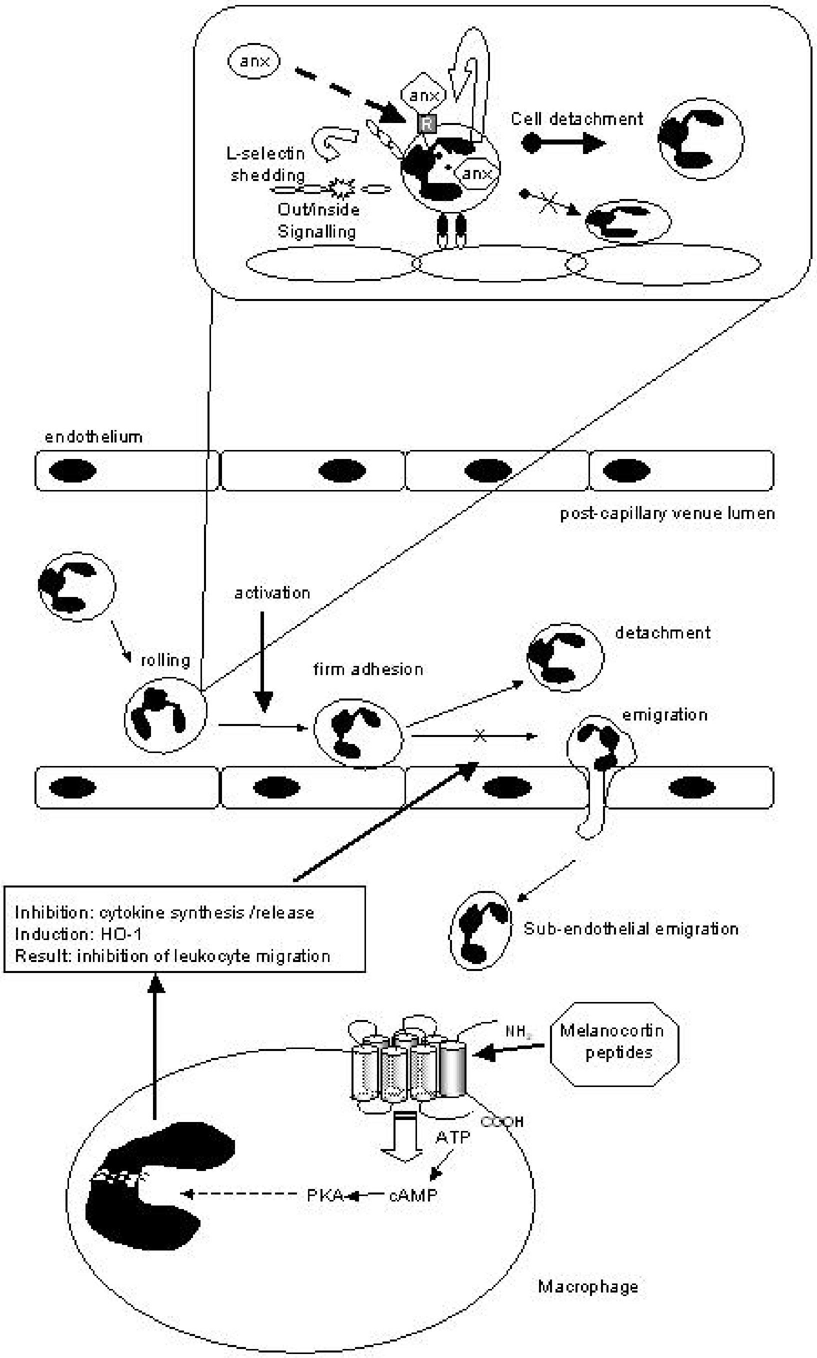

**FIGURE 3.** Schematic representation of the anti-inflammatory effects of the annexin and melanocortin peptides. Annexin 1 causes detachment of neutrophils from the endothelium by being released from gelatinase granules to the surface on activation of the cell. The detachment process could occur via two mechanisms: (1) the full-length protein could interact with receptor(s) to down-regulate neutrophil migration or (2) the protein is cleaved to make smaller pharmacologically active peptides that can also bind to the receptor and prevent neutrophil migration and also effect L-selectin shedding (highlighted in the smaller cartoon). The melanocortins display a different mechanism for modulating the host inflammatory response, resident MØ express MC3-R and agonism here leads to accumulation of cAMP with consequent activation of PKA. At least two major effects occur, with an early inhibition of release of proinflammatory cytokines/chemokines within the first 4 h and at later time-points, >4–6 h, the induction of the stress protein HO-1. Either mechanism of action can impact on the host inflammatory response and be responsible for the potent and reproducible antimigratory effects that melanocortin peptides exert in the context of acute local and systemic inflammation. The net effect with these endogenous peptides is that they modulate the host inflammatory response leading to a homeostatic balance within the tissue.

## CONCLUDING COMMENTS

The generation of proinflammatory cytokines and the associated inflammatory response are part of protective mechanisms initiated by the host to counteract insults and foreign pathogens, and reinstall tissue and organ homeostasis. Sometimes, though, the inflammatory process is more detrimental than life saving, as in the case of chronic inflammatory pathologies, including rheumatoid arthritis and inflammatory bowel disease[102]. This can be particularly true also for the tissue injury that follows IR of the heart[6,103]. The inflammatory response that ensues has the ultimate scope of resolving the damage, however it predisposes to tissue remodelling[15,104,105]. Targeting endogenous modulators of inflammation such as annexin 1 and the melanocortins could be an exciting avenue for future drug discovery. These peptides display distinct mechanisms of action, although both exert a protective effect. Annexin 1 prevents the circulating neutrophil from adhering to the endothelium and thus inhibiting its migration into tissue, whilst the melanocortins prevent activation of the endothelium by switching off the production of proinflammatory mediators from resident cells.

These compounds might have a potential advantage over existing therapies in that they may dampen down the host's response to inflammation, infection, and ischaemia, and play a protective role in maintaining a homeostatic balance within the body. Given their multitudes of action, in some respects they act like a "sledgehammer"; like a steroid, but without the side effects. It is always difficult to postulate whether peptide molecules could be first-line therapeutics due to the rapid clearance and moderately short half-lives. Although this may be a potential problem, they will have the advantage in the fact that they will not accumulate and some of the side effects associated with conventional treatments may be avoided[68]. In the future, new receptors, more potent and longer-lasting derivatives may be discovered. Another exciting opportunity may be in the development of dual inhibitors, such that the annexin 1 portion could be used to inhibit the rolling stage of the leukocyte, whilst the melanocortin portion will prevent the endothelium from becoming sticky. The net result would be inhibition of leukocyte migration to the site of tissue injury. Based on this, the potential use of lower doses of compounds might exist and, therefore, reduction of potential side effects might occur. What is clear is that we are entering an *era* where promoting the body's own natural defences could lead to novel therapeutics. Fig. 3 highlights the mechanism of action for these endogenous anti-inflammatory peptides.

## ACKNOWLEDGEMENTS

SJG, Senior Lecturer, is supported by the Research Advisory Board, St. Bartholomew's and the Royal London School of Medicine. (Grant Code RAB04/PJ/4), FNEG is a Fellow of the British Heart Foundation (Grant Code FS/03/100/16326).
